# Editorial: Common pathways in neurodevelopmental conditions and neurodegenerative disorders

**DOI:** 10.3389/fnins.2024.1454384

**Published:** 2024-08-07

**Authors:** Idris Long

**Affiliations:** School of Health Sciences, Health Campus, Universiti Sains Malaysia, Kubang Kerian, Kelantan, Malaysia

**Keywords:** neural tube diseases, transcription factor Egrl 1, GADD45 family protein, NAPB gene mutation, neurodegenerative disease

In the twenty-first century, research on neurodegenerative diseases has advanced at an accelerated rate. This compilation covers the following topics: the contribution of the growth arrest and DNA damage inducible protein 45 (GADD45) family protein on DNA demethylation; the impact of NSF Attachment Protein Beta (NAPB) gene mutations on early-onset epilepsy and autism; the combination of bibliometric and bioinformatics analysis on Neural Tube Defect (NTD) and the role of the EGR1 gene on various neurological processes. This compilation aims to educate people about the effects of the increasing incidence of neurodegenerative diseases. GADD45 family according to the Huang et al. is made up of stress-induced nuclear proteins that work with DNA demethylases to facilitate DNA demethylation. By modifying the expression patterns of specific genes, this process regulates a variety of cellular processes, including oxidative stress, DNA damage repair, apoptosis, proliferation, differentiation, inflammation, and neuroplasticity. This review delves deeply into the expression patterns and possible mechanisms of action linked to each member of the GADD45 family (GADD45α, GADD45β, and GADD45γ) in neurodevelopmental, neurodegenerative, and neuropsychiatric disorders. It also looks at ways to use these mechanisms for neurodegenerative intervention and treatment. Homozygous recessive genetic variant called NAPB gene mutations according to Ali et al., are the cause of early-onset epilepsy and autism. Primarily expressed in the brain, NAPB contributes to synaptic vesicle recycling by acting as a co-factor for NSF ATPase in the process of SNARE complex disassembly. All three afflicted children and their non-affected parents were used to create induced pluripotent stem cell (iPSC) lines, which differentiated into cortical neurons. Cortical neurons from parents and affected children were compared using electrophysiological and transcriptome methods. The results show that loss of NAPB function leads to changes in neuronal functions and most likely played a role in the triplets' poor neurodevelopment. Interestingly the NAPB genetic variation was repaired in two children iPSC by CRISPR/Cas9 gene editing. According to Cao et al., most of the research on neural tube defects (NTDs) is focused on molecular mechanisms of NTDs, such as genes and signaling pathways related to folate metabolism, neurogenic diseases caused by neural tube closure disorders, such as myelomeningocele and spina bifida, and prevention and treatment strategies, such as folate supplementation and surgical procedures. These findings were obtained through bibliometric combined with bioinformatics analysis. Most genes linked to NTDs are involved in development, part of cell projection, and molecular binding. These genes are primarily associated with signaling pathways such as PI3K-Akt, MAPK, wnt, and cancer. Chromosomes 1, 3, 5, 11, 14, and 17 have a significantly concentrated distribution of SNPs connected to non-typable dwarfism (NTDs), which could be linked to a higher chance of developing NTDs. Swilley et al. research focused on the transcription factor Egrl 1, which is a pivotal gene implicated in brain development and neuronal activity, and it has been linked to neurological disorders. Conditional Egrl 1 knockout was used in this investigation on mice of both sexes. Conditional knockout had no effect on anxiety or nociceptive response, but increased activity levels in females. In comparison to male mice, female mice also showed reduced recall ability after contextual fear learning. The analysis of RNA-seq data demonstrated that Nestin-Cre influences the expression of genes in the hippocampus. In Nestin-Cre mediated Egr1 conditional knockout mice, sex-related differences were magnified, and female mice are more susceptible to Egr1 gene loss. The control of the Wnt signaling pathway, extracellular matrix, and axon guidance were highly correlated with differentially expressed genes that emerged from the deletion of Egr1 in the neuronal cell lineage. They proposed that the deletion of Egr1 in the neuronal cell lineage and Nestin-Cre have different, sex-specific effects on hippocampus gene expression. In summary, the century-long progress in technology has enabled researchers to employ a wider range of sophisticated tools, techniques, and approaches to better understand the causes and manage numerous neurodevelopmental and neurodegenerative illnesses. I hope we can quickly have a definitive inverter to address this Research Topic.

## Author contributions

IL: Conceptualization, Writing – original draft.

